# Sex-Specific Effect of High-Fat Diet on Glycerol Metabolism in Murine Adipose Tissue and Liver

**DOI:** 10.3389/fendo.2020.577650

**Published:** 2020-10-21

**Authors:** Francesco Maria Iena, Johanne Blanner Jul, Jens Bay Vegger, Andreas Lodberg, Jesper Skovhus Thomsen, Annemarie Brüel, Janne Lebeck

**Affiliations:** Department of Biomedicine, Health, Aarhus University, Aarhus, Denmark

**Keywords:** aquaglyceroporins, adipose tissue, liver, Liraglutide, sex differences

## Abstract

Obesity is associated with increased plasma glycerol levels. The coordinated regulation of glycerol channels in adipose tissue (AQP7) and the liver (AQP9) has been suggested as an important contributor to the pathophysiology of type-2-diabetes mellitus, as it would provide glycerol for hepatic synthesis of glucose and triglycerides. The regulation of AQP7 and AQP9 is influenced by sex. This study investigates the effect of a high-fat diet (HFD) on glycerol metabolism in mice and the influence of sex and GLP-1-receptor agonist treatment. Female and male C57BL/6JRj mice were fed either a control diet or a HFD for 12 or 24 weeks. Liraglutide was administered (1 mg/kg/day) to a subset of female mice. After 12 weeks of HFD, females had gained less weight than males. In adipose tissue, only females demonstrated an increased abundance of AQP7, whereas only males demonstrated a significant increase in glycerol kinase abundance and adipocyte size. 24 weeks of HFD resulted in a more comparable effect on weight gain and adipose tissue in females and males. HFD resulted in marked hepatic steatosis in males only and had no significant effect on the hepatic abundance of AQP9. Liraglutide treatment generally attenuated the effects of HFD on glycerol metabolism. In conclusion, no coordinated upregulation of glycerol channels in adipose tissue and liver was observed in response to HFD. The effect of HFD on glycerol metabolism is sex-specific in mice, and we propose that the increased AQP7 abundance in female adipose tissue could contribute to their less severe response to HFD.

## Introduction

Obesity is a key risk factor for developing type-2-diabetes mellitus (T2D). The risk for developing T2D is sex-dependent and sex-related differences in triglyceride (TG) metabolism and body fat distribution clearly contribute to this (1). The molecular mechanisms behind the sex-dependent risk for developing T2D remain largely undetermined. Glycerol is stored as the backbone of TG, predominantly in adipose tissue, and it is released along with fatty acids in response to e.g. fasting and exercise. Obesity is associated with increased plasma glycerol levels (2). The coordinated upregulation of glycerol channels in white adipose tissue (WAT) and the liver has been suggested as an important contributor to the pathophysiology of T2D, as it would support the increased hepatic synthesis of glucose and TG (3, 4). In adipose tissue, the glycerol channel aquaporin 7 (AQP7) facilitates the efflux of glycerol (5). In male AQP7 knockout mice, increased activity of glycerol kinase (GlyK) in WAT has been reported alongside increased storage of triglyceride (6), which in turn led to obesity and secondary development of T2D (6). This suggests that controlling the availability of glycerol in adipose tissue is important for controlling TG accumulation in WAT. The glycerol released from WAT is primarily utilized in the liver, where the glycerol channel aquaporin 9 (AQP9) facilitates its uptake. In the liver, glycerol is utilized for gluconeogenesis (7, 8), glycolysis, and synthesis of acylglycerols including TG (9).

In different mouse models of obesity, a coordinated upregulation of AQP7 and AQP9 mRNA has been reported in male mice (3, 10). However, further studies are needed to confirm that the coordinated upregulation of AQP7 and AQP9 in relation to obesity also occurs at the protein level. Moreover, previous studies have shown that the expression of AQP7 in WAT (11, 12) and AQP9 in the liver (13, 14) is influenced by sex. Women respond to exercise training with an increased abundance of AQP7 in WAT, while men respond with a decreased abundance of AQP7 in WAT (11). In rats, increased hepatic abundance of AQP9 is found in response to starvation in males and ovariectomized females, whereas in intact females no increase is observed (13). These results indicate that the effect of obesity on glycerol metabolism could be sex-specific.

Liraglutide is a glucagon-like peptide-1 receptor (GLP-1R) agonist that is used to treat T2D and obesity (15). Its effects are not fully elucidated, but it augments insulin secretion, inhibits glucagon secretion, inhibits gastric emptying, and lowers food intake (16). The effect of GLP-1R agonists on glycerol metabolism remains to be investigated.

In the present study, we aim to investigate whether HFD-induced obesity in mice results in a coordinated upregulation of AQP7 and AQP9 protein in WAT and the liver and whether the effect of HFD on glycerol metabolism is sex-specific. Furthermore, we aim to investigate whether sex-differences in glycerol metabolism is likely to contribute to the sex-specific risk for developing insulin resistance in response to HFD in mice. Finally, here investigate whether Liraglutide treatment modulates glycerol metabolism in adipose tissue and the liver in female mice.

## Materials and Methods

### Animals

**Figure 1:**
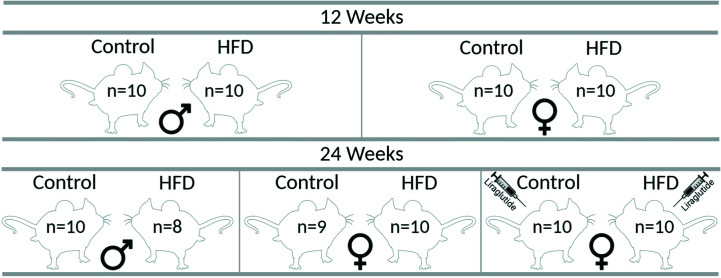
Graphic illustration of the different groups in the study. The indicated number of animals in each group correspond to the number of animals at the termination of the experiment, each group initially consisted of 10 mice each. The figure was created with BioRender.com.

Male (*n* = 40) and female (*n* = 60) C57BL/6JRj mice (Janvier Labs) were included in the study. Throughout the study period, the mice had free access to food and water and were kept with a 12:12-h artificial light-dark cycle, a temperature of 21°C ± 2°C, and a humidity of 55 ± 2%. At 16 weeks of age, animals of both sexes were, as illustrated in **Figure 1**, divided into 10 groups (*n* = 8–10 in each group at the termination of the experiment) receiving either a control diet containing 10% kilocalories (kcal) from fat, 20% kcal from protein and 70% kcal from carbohydrates with an energy density of 3.82 kcal/g (D12450J, Research Diets) or a HFD containing 60% kcal from fat, 20% kcal from protein and 20% kcal from carbohydrates with an energy density of 5.21 kcal/g (D12492, Research Diets) for 12 or 24 weeks. After 12 weeks of either control or HFD, one control group and one HFD group of female mice received Liraglutide (Novo Nordisk) s.c. (1 mg/kg/day) for the last 12 weeks before euthanasia. The mice received 0.1 mg/kg/day and 0.3 mg/kg/day of Liraglutide on days 1 and 2, respectively, before the full dosage was administered for the remaining part of the study. Body weight (BW) was determined every week and blood glucose levels (BG) every other week after 4–6 h of fasting using a glucometer (Accu-Chek Aviva, Roche) in the mice treated with diet for 24 weeks, as well as in the mice treated with diet for 12 weeks at the termination of the experiment. At the termination of the experiment, the animals were anesthetized using isoflurane and a large laparotomy was performed. Blood was collected from the abdominal aorta and perigonadal white adipose tissue, and the liver was removed for western blotting and paraffin embedding. The experimental protocol was approved by the Animal Experiments Inspectorate (2018–15–0201–01436) under the Ministry of Food, Agriculture and Fisheries, and in accordance with the guiding principles of the European Communities Directive of 22th of September 2010 (2010/63/EU) for animal experiments and the ARRIVE guidelines for reporting *in vivo* experiments (17).

### Semiquantitative Immunoblotting

Tissues from four to five mice from each group were analyzed using immunoblotting. The collected tissues were homogenized in dissection buffer and prepared for immunoblotting as previously described (13). The samples were loaded on AnykD Criterion TGX Precast Midi Protein gel (Bio-Rad) before separated by SDS-PAGE. The initial gels were stained using Gelcode Coomassie Blue Stain Reagent (Thermo Scientific) to adjust protein loading. Separated proteins were transferred to a PVDF membrane using the Trans-Blot Turbo transfer system (Bio-Rad) and antibody labeling was performed as previously described (13) using the following antibodies: AQP7 [RA2900/1246, (18)], ATGL (#2138, Cell Signaling Technology), HSL (#4107, Cell Signaling Technology), GlyK (ab126599, Abcam), AQP9 [RA2674-685, (19) [to ensure that the right bands were included in the semi-quantification for AQP9, the antibody was tested on a sample from an AQP9 knockout mouse (**Supplementary Figure 2**)], cGPDH (HPA044620, Sigma Aldrich), PLIN2 (#GP40, Progen), FATP2 (20) and FATP5 (21), Actin (A2066, Sigma Aldrich/Merck). After washing with PBS-T, the membranes were incubated with horseradish peroxidase-conjugated secondary antibodies (Dako A/S). Antibody binding was visualized by the use of ECL (Amersham Biosciences) and ImageQuant LAS 4000 (GE Healthcare). Band intensities were measured within the linear range using ImageJ software as outlined in the ImageJ documentation. Semiquantification was performed after background subtraction, and each lane was normalized to the results obtained from Coomassie staining (WAT) (**Supplementary Figure 1**) or actin (liver). In order to have quantifiable bands the intensity of the bands were equally adjusted by the brightness/contrast tool in ImageJ in some of the obtained membrane-images.

### Picro Sirius Red Stain and Immunohistochemistry

Tissues were immersion fixed for 24 h in 4% paraformaldehyde in PBS upon collection. After fixation, samples were dehydrated and embedded in paraffin wax. Then, 2-µm-thick sections were cut using a rotary microtome (Leica) and rehydrated before staining. *Picro-Sirius Red Stain:* After rinsing in tap water, sections were stained with Harris Hematoxylin (Merck) followed by rinsing in tap water and incubation with Picro-Sirius red dye (0.1% Sirius Red (RAL diagnostics) saturated with Picric Acid (Merck)) for up to 1 h. After washing, the sections were dehydrated and coverslips were mounted with Eukitt (Sigma). The obtained sections were scanned using a NanoZoomer S360 Digital slide scanner (Hamamatsu) and visualized with the Hamamatsu NDP.view2 Viewing software (U12388-01). Immunohistochemistry was carried out using indirect immunoperoxidase staining and performed as previously described (22) using the anti-AQP9 (RA2674-685) antibody. Microscopy was performed using a Leica DMRE light microscope.

### Quantification of Adipocyte Size

Relative adipocyte size was quantified using the scanned Picro-Sirius Red stained adipose tissue sections and the ImageJ plug-in Adiposoft (23). Pictures of the whole tissue section were acquired at a resolution of 7680 × 4840 pixels and subsequently increased in intensity in order to make adipocytes recognition easier for the plug-in. Areas were calculated considering the area per pixel present in each picture taken at a given magnification and resolution and expressed as μm^2^. Adipocytes with a minimum diameter of 2 μm and a maximum diameter of 250 μm were included. Each section was individually evaluated in order to remove areas that were wrongly assigned to be an adipocyte by the software.

### Plasma Measurements

Plasma glycerol and triglyceride levels (corrected for free-glycerol) were measured using a serum triglyceride determination kit (TR0100, Sigma-Aldrich/Merck).

### Statistical Analysis

GraphPad Prism was used for statistical calculations. The data are expressed as mean ± SEM and analyzed using ANOVA. The effect of HFD, Liraglutide or sex between control and HFD-fed animals was evaluated with Two-way ANOVA. One-way repeated-measures ANOVA was used to evaluate intragroup weight gain differences at each time point.

## Results

### Effect of HFD and Liraglutide Treatment on Body Weight on Blood Glucose Levels

**Figure 2:**
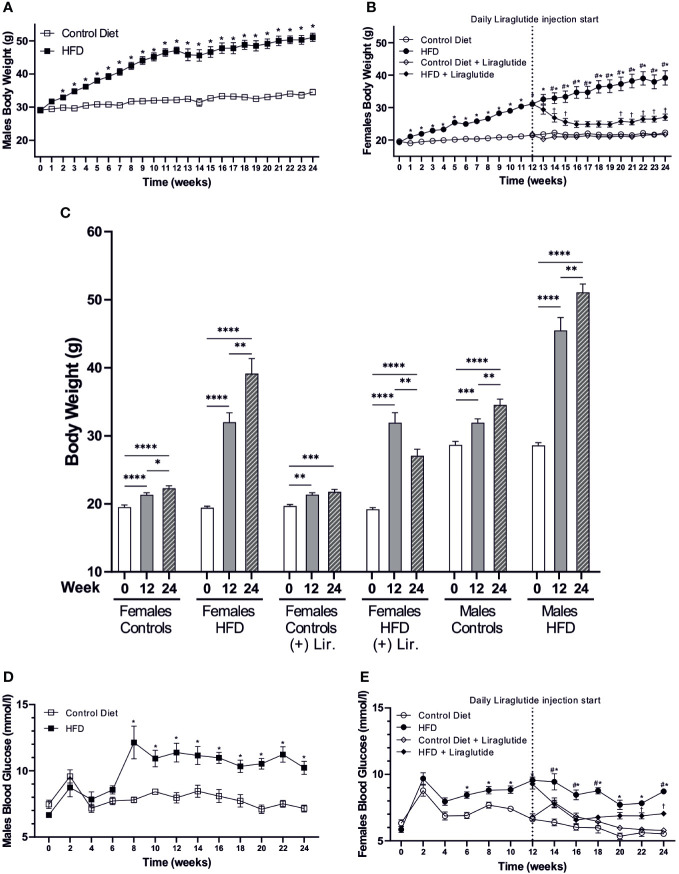
Body weight and blood glucose levels. **(A)** Body weight of high-fat diet (HFD) fed male mice compared to mice fed the control diet (*n* = 20 in each group, in the first 12 weeks, and for the remaining period *n* = 8–10). **(B)** Body weight of female mice fed the control or the HFD and female mice treated with Liraglutide in addition to being fed either the control or the HFD (*n* = 30 in each group in the first 12 weeks, and for the remaining period *n* = 9-10). **(C)** Body weight of female mice fed either the control or the HFD with or without Liraglutide treatment and male mice fed either the control or the HFD at baseline (0), 12 weeks (12) and 24 weeks (24) (*n* = 8–10 in each group). **(D)** Blood glucose levels in male mice fed either control or HFD (*n* = 8–10 in each group). **(E)** Blood glucose levels in female mice fed the control or the HFD or female mice treated with Liraglutide in addition to being fed either the control or the HFD (*n* = 19-20 for the first 12 weeks and hereafter 9-10 in each group). Values are expressed as mean ± SEM and * indicates statistical significant difference between HFD-fed animals and respective controls, # indicates statistical significant difference between HFD fed female mice with and without Liraglutide treatment, † indicates statistical significant difference between Liraglutide treated control mice and Liraglutide treated HFD-fed female mice. In **(C)** asterisks indicate significant differences between timepoints, *p < 0.05, **p < 0.01, ***p < 0.001, ****p < 0.0001.

Following 12 weeks of HFD, the BW was increased by 9 g or 40% in females (*p* < 0.0001) and by 15 g or 46% in males (*p* < 0.0001) compared to mice fed the control diet. After 24 weeks of HFD, the BW was increased by 17 g in both male and female mice compared to controls (*p* < 0.0001 for both), which corresponds to a 75% increase in females and a 48% increase in males (**Figures 2A, B**). Similarly, when evaluating the weight at Baseline, 12 and 24 weeks in each of the 24 weeks groups, the weight gain after 12 weeks of HFD was 12 g or 56% in females and 17 g or 62% in males. After 24 weeks of HFD, females had added a total of 20 g to their BW, whereas males had added 23 g, corresponding to a BW gain of 102% and 79%, respectively (**Figure 2C**). Liraglutide treatment reduced the BW of HFD-fed female mice after 2 weeks of treatment (*p* < 0.05), and after 4 weeks of treatment, the BW plateaued, resulting in a more modest 25% increase in BW after 24 weeks of HFD (p < 0.01) (**Figure 2B**). Despite the lower absolute weight gain in females after 12 weeks of HFD, both males (7.9 ± 0.4 vs 11.4 ± 0.7 mmol/l, *p* < 0.0001) and females (6.6 ± 0.2 vs 9.5 ± 0.2 mmol/l, *p* < 0.0001) demonstrated a significant increase in blood glucose (BG) levels compared to control fed mice. Similarly, after 24 weeks of HFD, both males (7.2 ± 0.3 vs 10.2 ± 0.5 mmol/l, *p* < 0.0001) and females (5.5 ± 0.1 vs 8.7 ± 0.1 mmol/l, *p* < 0.0001) had increased BG levels (**Figures 2D, E**). Treatment with Liraglutide significantly reduced the BG level of HFD-fed female mice after 2 weeks of treatment resulting in a more modest increase in the BG level at the termination of the experiment (5.8 ± 0.2 vs 7.1 ± 0.1 mmol/l, *p* < 0.01) compared to Liraglutide treated control females (**Figure 2E**).

### Sex-Specific Effect of HFD on AQP7 Abundance in White Adipose Tissue

**Figure 3:**
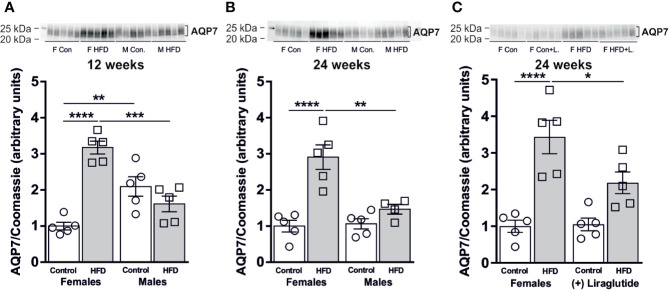
AQP7 protein abundance in adipose tissue from female and male mice after 12 and 24 weeks of high-fat diet (HFD) and in female mice after 12 weeks of Liraglutide treatment. Immunoblots and results of the densitometric analysis of the immunoblots for **(A)** AQP7 in adipose tissue from female and male mice after 12 weeks of either control or HFD. **(B)** AQP7 in adipose tissue from female and male mice after 24 weeks of either control or HFD. **(C)** AQP7 in adipose tissue from female mice fed either control or HFD and with or without Liraglutide treatment. Each lane represents one mouse. Band densities were normalized to total protein as evaluated by Coomassie blue staining. Mean band densities (*n* = 4–5) are presented relative to female controls. Asterisks indicate significant differences between groups, *p < 0.05, **p < 0.01, ***p < 0.001, ****p < 0.0001.

In order to evaluate the effect of diet-induced obesity (DIO) on WAT glycerol metabolism, we used immunoblotting to quantify the relative abundance of the glycerol channel AQP7. After 12 weeks of HFD, a significant 3.1-fold increase in AQP7 abundance was found in female mice compared to female mice fed the control diet (*p* < 0.0001). In contrast, no significant difference in WAT AQP7 abundance was observed between control and HFD-fed male mice (*p* = 0.35) (**Figure 3A**). The abundance of AQP7 in male control mice was 2.1-fold higher, than in female control mice (*p* < 0.01) (**Figure 3A**). A similar pattern was observed after 24 weeks of HFD, where a 2.9-fold increase in AQP7 abundance was observed in WAT from HFD-fed female mice (*p* < 0.0001), whereas no effect of HFD on AQP7 abundance was found in male mice (*p* = 0.60) (**Figure 3B**). Treatment with Liraglutide attenuated the increase in WAT AQP7 abundance observed in HFD-fed female mice by 36% (*p* < 0.05) and the abundance of AQP7 did not significantly differ between Liraglutide treated control and HFD-fed female mice (*p* = 0.07) (**Figure 3C**).

### HFD Decreases the Abundance of Lipolytic Enzymes in White Adipose Tissue

**Figure 4:**
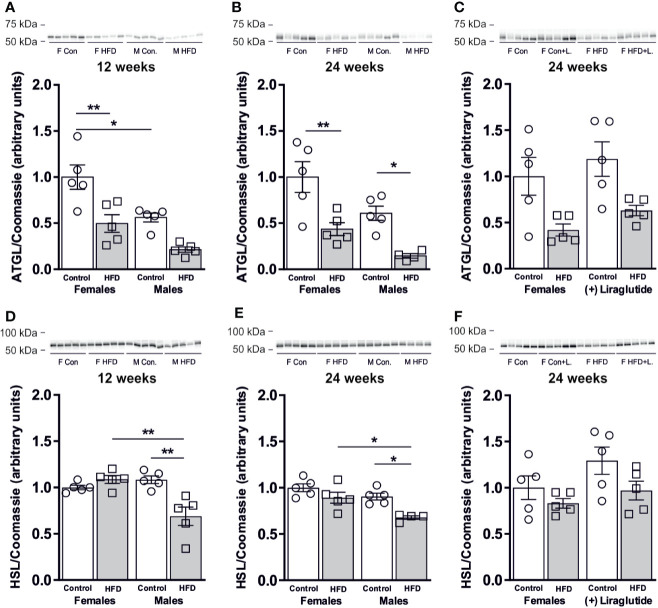
Protein abundance of lipases in adipose tissue from female and male mice after 12 and 24 weeks of high-fat diet (HFD) and in female mice after 12 weeks of Liraglutide treatment. Immunoblots and results of the densitometric analysis of the immunoblots for **(A)** Adipose triglyceride lipase (ATGL) in adipose tissue from female and male mice after 12 weeks of either control or HFD. **(B)** ATGL in adipose tissue from female and male mice after 24 weeks of either control or HFD. **(C)** ATGL in adipose tissue from female mice fed either control or HFD and with or without Liraglutide treatment. **(D)** Hormone sensitive lipase (HSL) in adipose tissue from female and male mice after 12 weeks of either control or HFD. **(E)** HSL in adipose tissue from female and male mice after 24 weeks of either control or HFD. **(F)** HSL in adipose tissue from female mice fed either control or HFD and with or without Liraglutide treatment. Each lane represents 1 mouse. Band densities were normalized to total protein as evaluated by Coomassie blue staining. Mean band densities (*n* = 4–5) are presented relative to female controls. Asterisks indicate significant differences between groups, *p < 0.05, **p < 0.01.

Next, we speculated whether the sex-specific increase in WAT AQP7 abundance in response to HFD would be associated with a sex-specific change in the WAT lipolytic rate. Adipose triglyceride lipase (ATGL) catalyzes the initial hydrolysis of TAG into diacylglycerol (DAG). After 12 weeks of HFD, female mice demonstrated a 50% reduction in the abundance of ATGL in WAT (*p* < 0.01). A similar trend was found in male mice after 12 weeks of HFD, however, this was not statistically significant (*p* = 0.05) (**Figure 4A**). When comparing mice fed the control diet, male mice had a 44% lower abundance of ATGL in WAT than female mice (*p* < 0.05) (**Figure 4A**). After 24 weeks of HFD, the abundance of ATGL was markedly reduced in response to HFD in both females (*p* < 0.01) and males (*p* < 0.05) (**Figure 4B**). Treatment with Liraglutide had no significant effect on the abundance of ATGL in WAT as compared to the nontreated HFD fed females (*p* = 0.74) or the treated control fed females (*p* = 0.07) (**Figure 4C**). When analyzing the abundance of hormone sensitive lipase (HSL) that primarily catalyzes the conversion of DAG to monoacylglycerol (MAG), we found no effect on total protein abundance after 12 weeks of HFD in female mice (*p* = 0.73), whereas a significant reduction in HSL protein abundance was observed in male mice (*p* < 0.01) (**Figure 4D**). Thus, HFD-fed male mice had a lower HSL abundance than HFD-fed female mice (*p* < 0.01). A similar pattern was observed after 24 weeks of HFD, where only males had reduced abundance of HSL in WAT in response to HFD (*p* < 0.05) (**Figure 4E**) and the abundance of HSL in HFD-fed male mice was significantly lower than in HFD-fed female mice (*p* < 0.05). Liraglutide treatment had no effect on the protein abundance of HSL in female mice as compared to the nontreated HFD fed females (*p* = 0.82) or the treated control fed females (*p* = 0.22) (**Figure 4F**).

In all, these results suggest that the lipolytic capacity was not increased in the investigated WAT depot in response to HFD in either female or male mice.

### Effect of HFD and Liraglutide Treatment on the Abundance of GlyK in White Adipose Tissue

**Figure 5:**
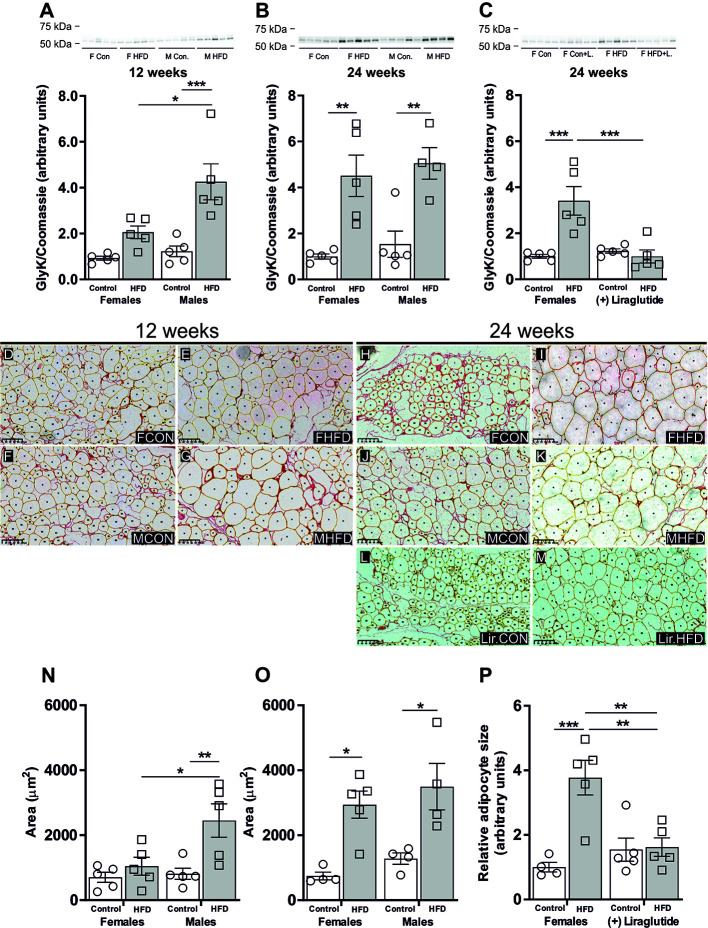
Glycerol kinase (GlyK) protein abundance in adipocyte size in adipose tissue from female and male mice after 12 and 24 weeks of high-fat diet (HFD) and in female mice after 12 weeks of Liraglutide treatment. Immunoblots and the results of the densitometric analysis of the immunoblots for **(A)** GlyK in adipose tissue from female and male mice after 12 weeks of either control or HFD. **(B)** GlyK in adipose tissue from female and male mice after 24 weeks of either control or HFD. **(C)** GlyK in adipose tissue from female mice fed either control or HFD and with or without Liraglutide treatment. Band densities were normalized to total protein as evaluated by Coomassie blue staining. Mean band densities (*n* = 4–5) are presented relative to female controls. **(D–G)** Representative images of adipose tissue sections stained with Picro-Sirius Red and analyzed by Adiposoft from male and female mice fed the control diet or the HFD for 12 weeks as indicated in the images. **(H–M)** Representative images of adipose tissue sections stained with Picro-Sirius Red and analyzed by Adiposoft from male and female mice (with or without Liraglutide treatment) fed the control or the HFD for 24 weeks as indicated in the images. **(N)** Mean adipocyte size in each group after 12 weeks of control or HFD (*n* = 5). **(O)** Mean adipocyte size in each group after 24 weeks of control or HFD (*n* = 4–5). **(P)** Effect of Liraglutide treatment on the relative adipocyte size in female mice fed control or HFD for 24 weeks (*n* = 4–5). Mean adipocyte areas are presented relative to female controls. Asterisks indicate significant differences between groups, *p < 0.05, **p < 0.01, ***p < 0.001.

The increased activity of glycerol kinase (GlyK) reported in male AQP7 KO mice likely results in increased glycerol-3-phosphate (G3P) availability in WAT, which promotes an increased accumulation of TG (6). Therefore, we wanted to evaluate whether the sex-specific regulation of AQP7 was correlated with a sex-specific regulation of GlyK. A marked 3.9-fold increase in the abundance of GlyK in WAT was found in male mice after 12 weeks of HFD (*p* < 0.001). A similar but less marked trend was observed in female mice, although it did not reach the level of statistical significance (*p* = 0.29). Consequently, HFD-fed male mice had a higher GlyK abundance than HFD-fed female mice (*p* < 0.05) (**Figure 5A**). After 24 weeks of HFD, both female and male mice demonstrated a marked increase in the abundance of GlyK in WAT (*p* < 0.01 for both) (**Figure 5B**). Liraglutide treatment normalized the abundance of GlyK in WAT in HFD-fed female mice (*p* < 0.001) (**Figure 5C**). Thus, after 12 weeks of HFD, the increase in WAT AQP7 abundance in female mice was associated with no significant increase in GlyK abundance, whereas in males the lack of increase in AQP7 was associated with a marked increase in the abundance of GlyK. After 24 weeks, the effect of HFD on WAT GlyK abundance was independent of sex. This could indicate that by increasing the AQP7 abundance, the HFD-fed female mice initially avoid an increased activity of GlyK in WAT, which leads to less accumulation of TG in WAT, whereas after 24 weeks, this is no longer sufficient to avoid activation of GlyK leading to increased TG accumulation.

### Sex-Specific Effect of HFD on Adipocyte Size

To test the above outlined hypothesis, we investigated the effect of HFD on adipocyte size. As illustrated in the representative images of the picro-sirius red stained adipose tissue from the different groups (**Figures 5D–M**), the size of the adipocytes varies and this heterogeneity increases in response to DIO. After 12 weeks of HFD, the mean adipocytes size in female mice was not significantly influenced by HFD (*p* = 0.83), whereas in male mice a 3.0-fold increase in mean adipocytes size was observed when compared to control fed mice (*p* < 0.01) (**Figure 5N**). After 24 weeks of HFD, female mice demonstrated a 4.0-fold increase in mean adipocyte size (*p* < 0.05), while males exhibited a 2.7-fold increase in mean adipocyte size (*p* < 0.05) (**Figure 5O**). Following Liraglutide treatment, the mean adipocyte size in HFD-fed female mice was reduced by 57% compared to non-treated HFD-fed females. Moreover, the mean adipocytes size did not differ between control and HFD-fed Liraglutide treated female mice (**Figure 5P**).

### Sex-Specific Effect of HFD on Plasma Glycerol and Triglyceride Levels

After 12 weeks of HFD, females tended towards having an increased plasma glycerol level (*p* = 0.05), whereas in males a significant increase was observed (*p* < 0.0001) (**Table 1**). After 24 weeks of HFD, the plasma glycerol level was significantly increased in both females (*p* < 0.05) and males (*p* < 0.01) (**Table 1**). Liraglutide treatment resulted in a normalization of the plasma glycerol levels in HFD-fed females, with no significant difference between control and HFD-fed Liraglutide-treated females (*p* = 0.30) (**Table 1**). The plasma triglyceride levels were increased in males after both 12 (*p* < 0.001) and 24 weeks (*p* < 0.01), while no significant increase was observed in females after both 12 (*p* = 0.66) and 24 weeks (*p* = 0.25) (**Table 1**).

**Table 1:** Serum glycerol, serum triglycerides (adjusted for free-glycerol), liver weight and liver weight normalized to bodyweight (BW) in female and male mice fed with control diet or HFD for 12 or 24 weeks or female mice treated with Liraglutide (+ L) in addition to being fed either the control diet or the HFD for 24 weeks.

	12 weeks of HFD													
	n	Glycerol (µM)	*p*	*p’*	Triglycerides (mM)	*p*	*p’*	n	Liver Weight (mg)	*p*		Liver weight/BW (%)	*p*	*p’*
Female Controls	10	129 ± 17			0.5 ± 0.1			10	841 ± 55			3.8 ± 0.2		
Females HFD	10	240 ± 40	0.05		0.6 ± 0.1	0.66		10	1017 ± 32	0.61		3.5 ± 0.1	0.66	
Male Controls	10	110 ± 15		0.97	0.6 ± 0.1		0.95	10	1198 ± 78			3.7 ± 0.2		0.97
Males HFD	10	349 ± 37	****	0.06	1.2 ± 0.1	***	***	10	2162 ± 174	****		4.4 ± 0.3	0.10	*
	**24 weeks of HFD**													
	**n**	**Glycerol** **(µM)**	***p***	***p’***	**Triglycerides** **(mM)**	***p***	***p’***	**n**	**Liver Weight** **(mg)**	***p***	***p’***	**Liver weight/BW** **(%)**	***p***	***p’***
Female Controls	7	86 ± 17			0.5 ± 0.1			9	861 ± 44			3.9 ± 0.2		
Female HFD	10	242 ± 43	*		0.8 ± 0.1	0.25		10	1206 ± 64	**		3.1 ± 0.1	**	
Male Controls	10	107 ± 16		0.97	0.9 ± 0.1		0.17	10	1435 ± 39			4.2 ± 0.1		0.71
Male HFD	7	319 ± 53	**	0.43	1.4 ± 0.2	**	**	8	2488 ± 136	****		4.9 ± 0.2	*	****
Female Controls + L	10	53 ± 12		0.88	0.5 ± 0.1		0.99	9	906 ± 40		0.99	4.2 ± 0.2		0.71
Females HFD + L	10	126 ± 30	0.30	*	0.6 ± 0.1	0.80	0.38	10	1077 ± 25	0.39	0.66	4.0 ± 0.1	0.96	***

* denotes statistical significant difference. *p < 0.05, **p < 0.01, ***p < 0.001, ****p < 0.0001. P-values are given in two columns. In the first column (p), the values indicate the comparison of same sex control and HFD groups. In the second column (p’) the values indicate the comparison of different sexes but same treatment groups (this was not evaluated for liver weight) or same sex with or without Liraglutide treatment.

### No Effect of HFD on the Hepatic Abundance of AQP9

**Figure 6:**
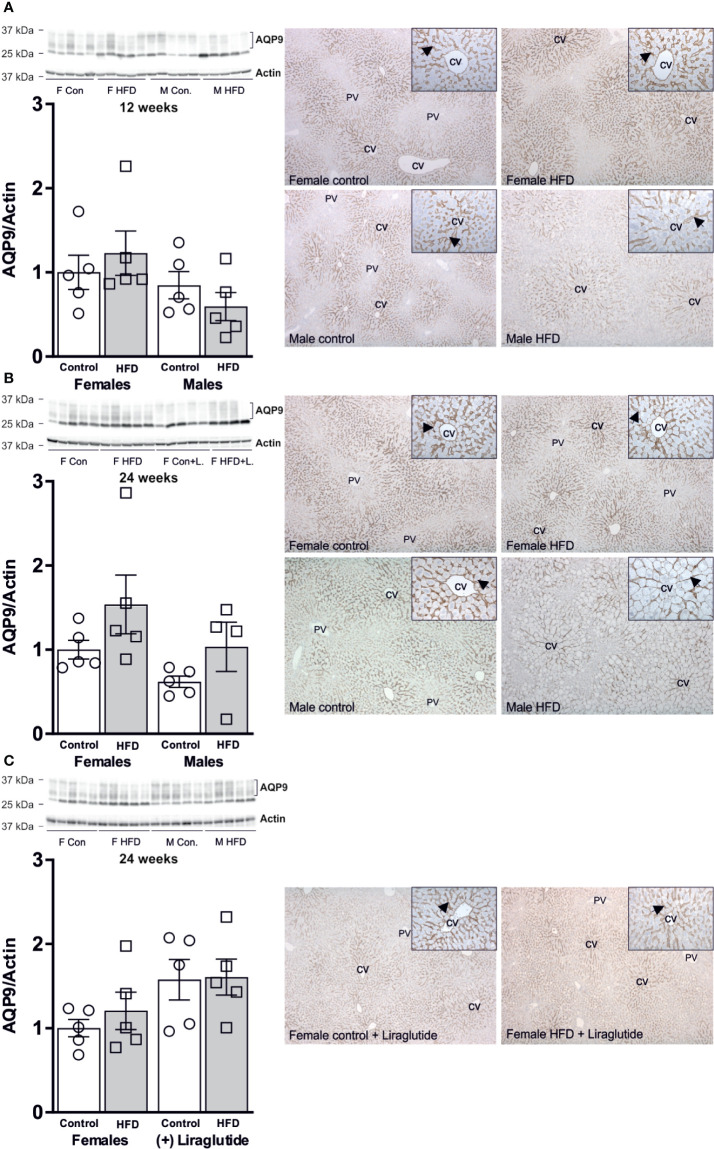
Hepatic AQP9 protein abundance in female and male mice after 12 and 24 weeks of high-fat diet (HFD) and in female mice after 12 weeks of Liraglutide treatment. Immunoblots and results of its densitometric analysis and representative images of immunoperoxidase labeling for **(A)** AQP9 in the liver from female and male mice after 12 weeks of either control or HFD. **(B)** AQP9 in the liver from female and male mice after 24 weeks of either control or HFD. **(C)** AQP9 abundance in the liver from female mice fed either control or HFD and with or without Liraglutide treatment. Each lane represents 1 mouse. Band densities were normalized to actin bands from the same membrane. Mean band densities (n = 4–5) are presented relative to female controls. Representative images of immunoperoxidase staining for AQP9 in the liver is shown at ×5 or ×25 (inserts) magnification. CV, central vein. PV, portal vein. Arrows indicate immunoperoxidase staining for AQP9 in the basolateral plasma membrane domain of hepatocytes in the centrilobular region.

As outlined above, the liver is the major organ responsible for utilizing released glycerol. After both 12 and 24 weeks of HFD, no significant effect on the hepatic abundance of the glycerol channel AQP9 was observed in either female (*p* = 0.86) or male mice (*p* = 0.82) (**Figures 6A, B**). Treatment with Liraglutide had no effect on the hepatic abundance of AQP9 in the control (*p* = 0.22) or HFD fed female mice (*p* = 0.52) (**Figure 6C**). Immunohistochemical analysis of hepatic AQP9 abundance is consistent with the results obtained using immunoblotting with no clear effect of HFD on the labeling for AQP9 in the basolateral plasma membrane domain of perivenous hepatocytes.

### Effect of HFD on the Hepatic Abundance of GlyK and cGPDH

**Figure 7:**
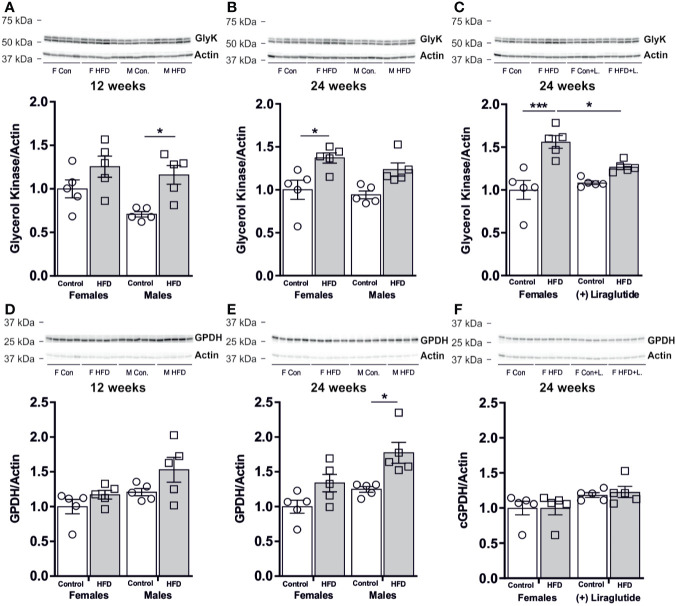
Hepatic protein abundance of GlyK and cGPDH in female and male mice after 12 and 24 weeks of high-fat diet (HFD) and in control or HFD-fed female mice after 12 weeks of Liraglutide treatment. Immunoblots and the results of the densitometric analysis of the immunoblots for **(A)** GlyK in the liver from female and male mice after 12 weeks of either control or HFD. **(B)** GlyK in the liver from female and male mice after 24 weeks of either control or HFD. **(C)** GlyK in the liver from female mice fed either control or HFD and with or without Liraglutide treatment. **(D)** cGPDH in the liver from female and male mice after 12 weeks of either control or HFD. **(E)** cGPDH in the liver from female and male mice after 24 weeks of either control or HFD. **(F)** cGPDH in the liver from female mice fed either control or HFD and with or without Liraglutide treatment. Each lane represents 1 mouse. Band densities were normalized to actin bands from the same membrane. Mean band densities (*n* = 4–5) are presented relative to female controls. Asterisks indicate significant differences between groups, *p < 0.05, ***p < 0.001.

After 12 weeks of HFD, male mice had a 64% increase in the hepatic abundance of GlyK compared to control fed mice (*p* < 0.05), whereas in females no significant difference was observed (*p* = 0.29) (**Figure 7A**). After 24 weeks of HFD, female mice had an increased hepatic abundance of GlyK (*p* < 0.05) and a similar trend was observed for male mice (*p* = 0.11) (**Figure 7B**). Liraglutide treatment attenuated the increase in GlyK found in females after 24 weeks of HFD (*p* < 0.05) (**Figure 7C**). The modest increase in GlyK abundance in response to HFD likely increases the hepatic synthesis of G3P that can be utilized for either gluconeogenesis or synthesis of lysophosphatidic acid (LPA), the initial step in TG synthesis. Cytosolic glycerol-phosphate-dehydrogenase (cGPDH) catalyzes the reversible conversion of G3P into dihydroxyacetone phosphate (DHAP) and is essential for channeling either G3P into gluconeogenesis or DHAP into G3P synthesis. The hepatic protein abundance of cGPDH remained unaffected after 12 weeks of HFD in both female (*p* = 0.70) and male mice (*p* = 0.21) (**Figure 7D**). After 24 weeks of HFD, the abundance of cGPDH was increased by 42% in male mice (*p* < 0.05), whereas in females no difference was observed (*p* = 0.17) (**Figure 7E**). Liraglutide treatment had no significant effect on the cGPDH protein abundance in the control (*p* = 0.40) or HFD fed females mice (*p* = 0.23) (**Figure 7F**).

### Hepatic Steatosis Is Induced by HFD Mainly in Male Mice

After both 12 and 24 weeks of HFD, the images of hepatic AQP9 abundance also revealed a much more marked ballooning of the perivenous hepatocytes in HFD-fed male mice than in HFD-fed female mice (**Figure 6**). Ballooning reflects accumulation of fat and thus a higher degree of hepatic steatosis in HFD-fed males. In support, after 12 weeks of HFD, male mice increased their liver weight (LW) 1.8-fold (*p* < 0.0001), whereas no significant effect was observed in females (*p* = 0.61). After 24 weeks of HFD, male mice demonstrated a 1.7-fold increase in LW (*p* < 0.0001), whereas a more modest 1.4-fold increase was observed in female mice (*p* < 0.01) (Tabel 1). Liver weight was not significantly influenced by Liraglutide treatment in neither control (*p* = 0.99) or HFD (*p* = 0.66) fed females (Tabel 1). HFD-fed female mice demonstrated a lower LW/BW ratio than HFD-fed males both after 12 (*p* < 0.05) and 24 (*p* < 0.0001) weeks of HFD, suggesting that female mice are more prone to store fat in extra-hepatic tissues than males (Tabel 1). Treatment with Liraglutide normalized the LW/BW ratio in HFD-fed females (*p* < 0.001), which mainly reflects the effect of Liraglutide on body weight, as no significant difference in liver weight was observed between the two groups (Tabel 1). In all, this supports that male mice are more prone to develop hepatic steatosis than female mice in response to HFD.

### Effect of HFD and Liraglutide Treatment on the Abundance of PLIN2 in the Liver

**Figure 8:**
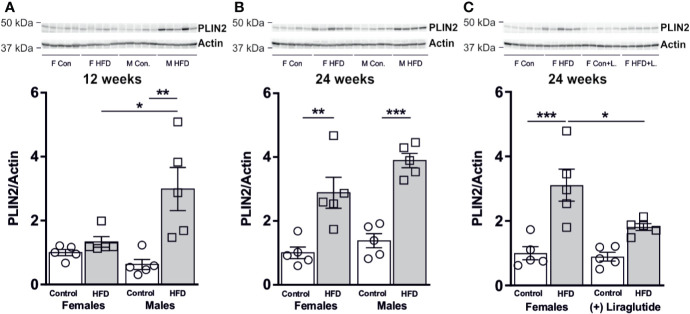
Hepatic PLIN2 protein abundance in male and female mice after 12 and 24 weeks of high-fat diet (HFD) and in control or HFD-fed female mice after 12 weeks of Liraglutide treatment. Immunoblots and the results of the densitometric analysis of the immunoblots for **(A)** PLIN2 in the liver from female and male mice after 12 weeks of either control or HFD. **(B)** PLIN2 abundance in female and male mice after 24 weeks of either control or HFD. **(C)** PLIN2 abundance in the liver from female mice fed either control or HFD and with or without Liraglutide treatment. Each lane represents 1 mouse. Band densities were normalized to actin from a different membrane, where the same amounts of the samples were loaded. Mean band densities (*n* = 4–5) are presented relative to female controls. Asterisks indicate significant differences between groups, *p < 0.05, **p < 0.01, ***p < 0.001.

The changes in liver weight were to some extent associated with an increased hepatic abundance of the lipid droplet associated protein perilipin 2 (PLIN2). After 12 weeks of HFD, male mice demonstrated a 4.8-fold increase in the hepatic abundance of PLIN2 (*p* < 0.01), whereas in female mice no differences were observed (*p* = 0.91) (**Figure 8A**). After 24 weeks, a similar increase in the abundance of PLIN2 was found in HFD-fed male (*p* < 0.001) and female mice (*p* < 0.01) (**Figure 8B**). Treatment with Liraglutide attenuated the increased abundance of PLIN2 in HFD females (*p* < 0.05) (**Figure 8C**).

### Sex-Specific Effect of HFD on the Hepatic Abundance of FATP2 and FATP5

**Figure 9:**
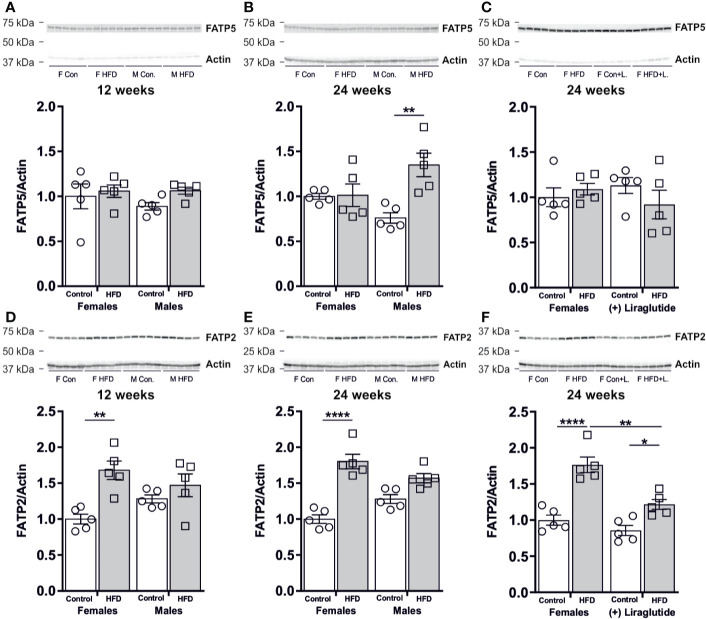
Hepatic protein abundance of fatty acid transport proteins after 12 and 24 weeks of high-fat diet (HFD) and in control or HFD-fed female mice after 12 weeks of Liraglutide treatment. Immunoblots and the results of the densitometric analysis of the immunoblots for **(A)** FATP5 abundance in the liver from female and male mice after 12 weeks of either control or HFD. **(B)** FATP5 abundance in the liver from female and male mice after 24 weeks of either control or HFD. **(C)** FATP5 abundance in the liver from female mice fed either control or HFD and with or without Liraglutide treatment. **(D)** FATP2 in the liver from female and male mice after 12 weeks of either control or HFD. **(E)** FATP2 in the liver from female and male mice after 24 weeks of either control or HFD. **(F)** FATP2 in the liver from female mice fed either control or HFD and with or without Liraglutide treatment. Each lane represents 1 mouse. Band densities were normalized to actin bands from the same membrane. Mean band densities (*n* = 4–5) are presented relative to female controls. Asterisks indicate significant differences between groups, *p < 0.05, **p < 0.01, ****p < 0.0001.

As hepatic steatosis was predominantly observed in male mice, we hypothesized that the hepatic uptake of fatty acids for triglyceride synthesis would be increased mainly in HFD-fed male mice. No effect on the protein abundance of the fatty acid transport protein 5 (FATP5) was observed in the liver after 12 weeks of HFD in female (*p* = 0.42) or male mice (*p* = 0.45) (**Figure 9A**). However, after 24 weeks of HFD, a 1.8-fold increase in the FATP5 protein abundance was observed in male mice (*p* < 0.01) (**Figure 9B**). No effect of Liraglutide on the hepatic abundance of FATP5 was observed (*p* = 0.69) (**Figure 9C**). By contrast, both 12 and 24 weeks of HFD, increased the hepatic abundance of the fatty acid transport protein 2 (FATP2) in female mice compared to control fed mice, whereas no significant effect of HFD was observed in male mice after 12 (*p* = 0.64) or 24 weeks (*p* = 0.06) (**Figures 9D, E**). Treatment with Liraglutide attenuated the effect of HFD on hepatic FATP2 abundance, when compared to the nontreated HFD fed females (*p* < 0.01) (**Figure 9F**).

## Discussion

In the present study, we examined the effect of 12 and 24 weeks of 60% HFD feeding in female and male mice on WAT and liver glycerol metabolism. In addition, the effect of Liraglutide treatment on glycerol metabolism was investigated in female mice. Similar to previous observations, we found that female mice are less prone to gain weight in response to HFD feeding compared to male mice (24–26). However, due to the lower body weight of female control mice, the relative weight gain only differed by 6% after 12 weeks of HFD. In a previous study of c57BL/6N mice fed either a 42% HFD (*n* = 586) or a control diet containing 21% kcal from fat (*n* = 733) for 16 weeks, male mice also only demonstrated a slightly higher relative body weight gain when compared to females. However, despite the modest difference in relative weight gain, it was demonstrated that the effect of HFD on the majority of parameters evaluated including glucose tolerance and glucose clearance was sex-specific with males generally demonstrating a more severe phenotype (27). This shows that even a minor difference in body weight gain is associated with a more severe response to HFD in male mice. However, after 24 weeks of HFD females demonstrated a more pronounced increase in relative body weight gain than males supporting that the relative resistance to diet-induced obesity (DIO) in females is attenuated after 24 weeks of HFD. This is somewhat in line with the study by Bruder-Nascimento and co-workers, where male and female C57Bl/6 mice had a similar BW after 24 weeks of 60% HFD feeding (28). However, despite a similar degree of adiposity after 24 weeks of HFD in male and female mice, then male mice still had a more severe phenotype that included more marked hyperglycemia, hypertriglyceridemia and a higher degree of hepatic steatosis. As expected (29), Liraglutide treatment markedly reduced BW and BG levels in female HFD-fed mice.

**Figure 10:**
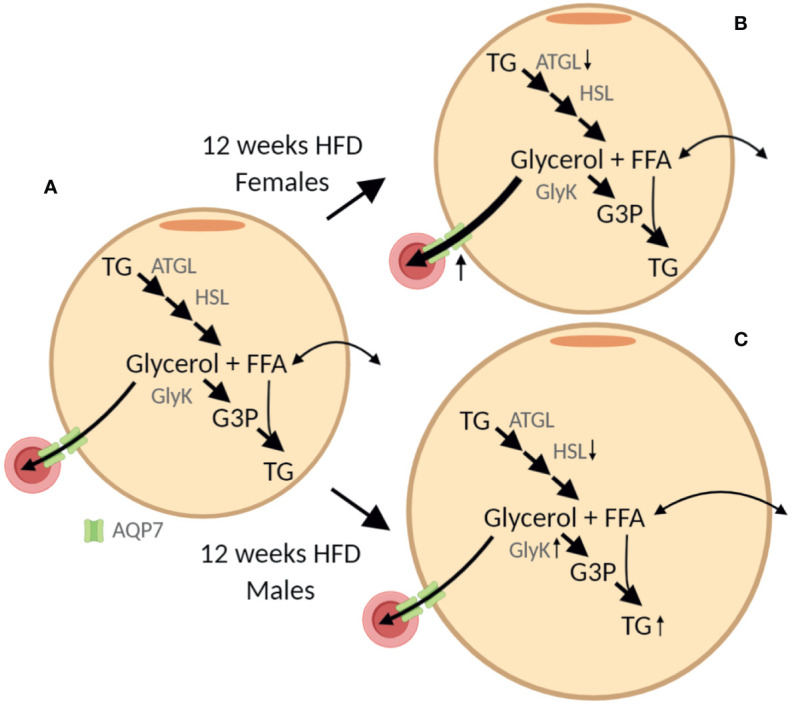
Graphic illustration of the sex-specific effect of 12 weeks of high-fat diet (HFD) on adipose tissue. **(A)** In the adipocyte triglyceride (TG) is stepwise hydrolyzed into glycerol and free fatty acids (FFA) by adipose triglyceride lipase (ATGL), hormone sensitive lipase (HSL) and monoacylglycerol lipase (not shown). The efflux of glycerol from adipose tissue is facilitated by aquaporin 7 (AQP7) expressed both in the adipocytes and the endothelial cells. Alternatively, glycerol can be phosphorylated into glycerol-3-phosphate (G3P) by glycerol kinase (GlyK) that can be reutilized for TG synthesis. Importantly, in healthy adipose tissue, the activity of GlyK is limited and the majority of glycerol is released for metabolism in other tissues. **(B, C)** From the results obtained after 12 weeks of HFD we hypothesize that, even though both female and male mice seem to have a reduced lipolytic rate per adipose tissue protein amount, the lack of increased AQP7 abundance in male mice results in accumulation of glycerol in the adipocytes, which in turn leads to increased abundance of GlyK and increased phosphorylation of glycerol into G3P. This in turn promotes the synthesis of TG and an increased accumulation of TG in the adipose tissue as indicated by the increased adipocyte size. The figure was created with BioRender.com.

The sex-specific effect of HFD was associated with a marked increase in AT AQP7 abundance in female mice only. The lack of a significant increase of WAT AQP7 abundance in male mice is partly contrasting previous observations, where an increase in WAT AQP7 protein has been reported in obese male ob/ob (30) and db/db (3) mice. However, since leptin has been shown to reduce the AT AQP7 abundance (30, 31) the effect of obesity in the setting of leptin/leptin receptor deficiency is difficult to interpret. To our knowledge, only one study has investigated the effect of DIO on WAT AQP7 abundance, where a modest increase in AQP7 mRNA was reported after 16 weeks of HFD in male mice (10). In a previous study, an association between obesity and a single-nucleotide-polymorphism in the promotor of AQP7 that causes a decreased transcription of the *aqp7* gene was found in women only (32). Moreover, we have previously shown that only women respond to exercise training with an increased abundance of AQP7 in WAT (11). Taken together, these findings, may suggest that a high abundance of AQP7 in WAT is particularly beneficial in females. In male AQP7 KO mice, increased activity of glycerol kinase was observed together with increased accumulation of TG in adipocytes (6). We, therefore, speculate that increased abundance of AQP7 in WAT could prevent an increased activity of glycerol kinase and lead to less accumulation of TG in AT as illustrated in **Figure 10**. Interestingly, we found that after 12 weeks of HFD, only male mice demonstrated an increased abundance of glycerol kinase and hypertrophy of adipocytes. However, after 24 weeks of HFD, both male and female mice have an increased abundance of glycerol kinase and hypertrophic adipocytes despite the increased AQP7 abundance in female AT.

Liraglutide treatment attenuated the effect of HFD on AT AQP7 and glycerol kinase abundance and normalized the adipocyte size. This outcome is likely, at least in part, due to an indirect effect of the weight loss as well as Liraglutide treatment is known to induce increased insulin secretion. Canonical GLP-1 receptors (GLP-1R) has not been detected in adipocytes (16, 33). However, AQP7 is in AT predominantly expressed in endothelial cells (22) and GLP-1R mRNA has been shown in the stromal vascular fraction from AT (33), and GLP-1R abundance has been reported in renal endothelial cells (34). Together, this may suggest that GLP-1R is expressed in endothelial cells in AT, thereby allowing a direct influence of Liraglutide on AQP7 abundance in WAT endothelial cells.

The reduced abundance of the AT lipase ATGL in response to HFD is consistent with previous observations (35, 36). Likewise, the reduction in AT HSL abundance in HFD-fed males is in alignment with results obtained in obese individuals (37, 38). Even though measurements of enzyme activity should have been applied to fully elucidate the lipolytic rate in WAT in this study, the findings in the present study are in line with the notion that even though the overall lipolytic activity is increased in response to obesity, the lipolytic activity per kilogram fat mass is actually reduced in obesity (39). Studies in male rodents have led to the general assumption that the AT AQP7 abundance is increased in states with increased lipolytic rate such as fasting (40). However, in fasted male mice increased abundance of AQP7 protein was only found after 72 h, but not after 24 h of fasting (22). This indicates that prolonged fasting/starvation is required before the WAT AQP7 protein abundance is increased in males. In female mice, no indications for an association between the HFD-induced increase in AT AQP7 abundance and increased WAT lipolytic activity was found as evaluated by the marked decrease in WAT ATGL abundance. In a recent study, we also found no evidence for a positive correlation between WAT AQP7 protein abundance and lipolytic rate in lean or obese T2D men (41). *In vitro* studies in adipocytes have suggested that trafficking of AQP7 to the plasma membrane occurs in response to lipolytic stimuli (42, 43). Even though the proposed dynamic localization of AQP7 in the adipocyte remains to be confirmed *in vivo*, further studies are needed to clarify if the effect of HFD on total protein abundance of AQP7 found in this study also involves changes in the subcellular localization of AQP7 in adipose tissue. In all, these findings indicate that WAT AQP7 protein abundance does not necessarily follow the WAT lipolytic rate. Instead, we here propose that the regulation of WAT AQP7 is centered around controlling the intra-adipose tissue glycerol concentration. We speculate, that HFD fed female mice initially maintains a lower intra-adipose tissue glycerol concentration by increasing the abundance of AQP7. Which in turn results in no increased in glycerol kinase activity and thus a lower availability of G3P for TG synthesis leading to less accumulation fat in adipose tissue. However, further studies are needed to test this novel hypothesis and unravel why the effect is not maintained after 24 weeks of HFD.

In humans, obesity is associated with increased plasma glycerol levels, where women have higher plasma glycerol levels than men (2). Here, we also find that HFD induces elevated plasma glycerol levels in both male and female mice. However, we also find a trend for males responding to HFD with a higher plasma glycerol level than females. After 12 weeks of HFD, this likely, at least in part, reflects the less marked absolute weight gain in females, whereas after 24 weeks of HFD, the absolute weight gain was similar in males and females and other mechanisms likely contribute. The liver is the major organ responsible for glycerol utilization (44), and the FFA and glycerol released as a result of WAT lipolysis are central drivers of the increased hepatic gluconeogenesis seen in T2D (45). AQP9 facilitates the hepatic uptake of glycerol for gluconeogenesis (7, 8, 46). As demonstrated by Jin and co-workers, glycerol is, in addition to being a precursor for glucose synthesis, also a significant contributor to the hepatic synthesis of acylglycerols including TG (9). Moreover, in male rats, 8 weeks of HFD was associated with an increased hepatic AQP9 abundance and knockdown of AQP9 was reported to alleviate the degree of hepatic steatosis (47). Thus, indicating that blocking AQP9 could be a mean to reduce hepatic synthesis of both TG and glucose, at least in rats. However, the hepatic TG content did not differ between obese db/db mice lacking AQP9 and wildtype db/db mice and the obese AQP9 KO mice even displayed a modestly elevated BG level compared to the wildtype db/db mice (48). Moreover, when comparing lean and leptin deficient ob/ob male mice in the fed state no difference in hepatic AQP9 abundance was reported (30), whereas in another study of 18 h fasted ob/ob mice a lower hepatic AQP9 abundance was shown (49). Like for AQP7, leptin has been shown to regulate hepatic AQP9 abundance (30, 31), thus complicating the interpretation of the effect of obesity on AQP9 abundance in leptin deficient mice. In our study, no significant effect of HFD on AQP9 protein abundance was observed. Even though there was a trend towards higher hepatic AQP9 abundance in response to 24 weeks of HFD in both sexes, the more marked hepatic steatosis in males after both 12 and 24 weeks of HFD was not associated with a higher abundance of AQP9 in males than in females. Furthermore, we found no evidence for a coordinated regulation of the protein abundance of AQP7 in WAT and AQP9 in the liver in mice as previously suggested (3).

HFD influenced the hepatic abundance of GlyK in a manner that supports an increased hepatic synthesis of G3P from glycerol that is initiated at an earlier timepoint in males. Furthermore, only males increased the hepatic abundance of cGPDH indicating either increased channeling of glycerol towards gluconeogenesis or synthesis of G3P from glucose or pyruvate. Overall, this indicates that the hepatic handling of glycerol in response to DIO is sex-specific and indicates that the development of hepatic steatosis is associated with increased hepatic glycerol metabolism.

We speculated that the degree of hepatic steatosis would be associated with an increased abundance of fatty acid transport proteins. Knockdown of FATP2 (20) and FATP5 (50) in HFD-fed mice reduces the degree of hepatic steatosis, thus demonstrating an important role in controlling hepatic TG accumulation. Here, the effect of HFD on hepatic FATP2 and FATP5 abundance was sex-specific and only male mice fed HFD for 24 weeks demonstrated an association between increased abundance of FATP5 and increased hepatic steatosis. In support of FATP5 being a more significant contributor to hepatic TG accumulation than FATP2 is the more pronounced effect of FATP5 knockdown (50) on hepatic steatosis than knockdown of FATP2 (20). Knockout of PLIN2 also alleviates diet induced hepatic steatosis (51, 52). Here we find that the increased hepatic PLIN2 abundance to some extent parallels the degree of hepatic TG accumulation. However, after 24 weeks of HFD, the increase in PLIN2 was similar in female and male mice, where females displayed a more modest degree of hepatic steatosis than males, which could indicate that increased abundance of PLIN2 is promoted already in the early phases of hepatic steatosis development. Liraglutide treatment normalized the increased abundance of GlyK, FATP2 and PLIN2 in HFD-fed female mice. This is likely secondary to the obtained weight loss and increased insulin secretion as previous studies have shown no abundance of the GLP-1R in hepatocytes (33).

The present study has some limitations. Firstly, the effect of Liraglutide was investigated in female mice only and since the effect of DIO is generally more pronounced in males, it would also have been interesting to determine the effect of Liraglutide on glycerol metabolism in male mice. Furthermore, we only investigated the total protein abundance of the different enzymes relevant for glycerol metabolism and lipolysis. An evaluation of the enzymatic activity would have been preferable or alternatively the phosphorylation status of e.g. HSL would have provide more information regarding the activity of the enzyme. Finally, the abundance of AQP7 in WAT was evaluated in whole adipose tissue and thus, does not provide any information about how the cellular (endothelial cells/adipocytes) or subcellular (intracellular/plasma membrane) abundance of the protein was affected. Despite these limitations, the study still provides novel information regarding the effect of HFD, sex, and Liraglutide treatment on glycerol metabolism in adipose tissue and the liver.

In conclusion, consistent with previous studies, we find that female mice are less susceptible to develop obesity than male mice in response to HFD feeding. However, the sex-differences becomes less marked after prolonged HFD feeding. The sex-specific response to HFD is associated with sex-specific regulation of the proteins involved in glycerol metabolism in WAT and the liver. We propose that the markedly elevated AQP7 abundance in female WAT after 12 weeks of HFD contributes to the less severe accumulation of TG in female WAT by facilitating an increased efflux of glycerol from WAT and thus less activation of glycerol kinase. No evidence for a coordinated upregulation of WAT AQP7 and liver AQP9 protein abundance in response to HFD was observed. Finally, treatment of HFD-fed females with Liraglutide generally attenuated the effects of HFD on glycerol metabolism in WAT and the liver, thus indirectly illustrating that changes in glycerol metabolism are indeed involved in the pathophysiology of insulin resistance.

## Data Availability Statement

‘The original contributions presented in the study are included in the article/**Supplementary Material**; further inquiries can be directed to the corresponding author.

## Ethics Statement

The animal study was reviewed and approved by the Animal Experiments Inspectorate (2018–15–0201–01436), under the Ministry of Food, Agriculture and Fisheries.

## Author Contributions

FI and JJ contributed to the design of the work, to the acquisition, analysis and interpretation of data, manuscript drafting and contributed to the writing of the manuscript. JV and AL contributed to the design of the work and to the acquisition of the experimental material. JT contributed to the design of the work and the data interpretation and provided suggestions for the manuscript draft. AB contributed to the design of the work, the acquisition of the experimental material and to the data interpretation and provided suggestions for the manuscript draft. JL contributed to the design of the work, to the acquisition of experimental material, the data interpretation, and the manuscript writing. All authors contributed to the article and approved the submitted version.

## Funding

FI was funded by Aarhus University Research Foundation and Health, Aarhus University. JJ and JL were funded by Aarhus University Research Foundation. JV and AL were funded by A.P. Moeller Foundation for the Advancement of Medical Science (grant number 15315) and Health, Aarhus University.

## Conflict of Interest

The authors declare that the research was conducted in the absence of any commercial or financial relationships that could be construed as a potential conflict of interest.
