# Supplementary material for: Sex-Specific Effect of High-Fat Diet on Glycerol Metabolism in Murine Adipose Tissue and Liver
Source: Front Endocrinol (Lausanne). 2020 Oct 21;11:577650. doi: 10.3389/fendo.2020.577650 (PMC7609944; doi:10.3389/fendo.2020.577650)
Supplement: Supplementary file 1 [file DataSheet_1.docx]

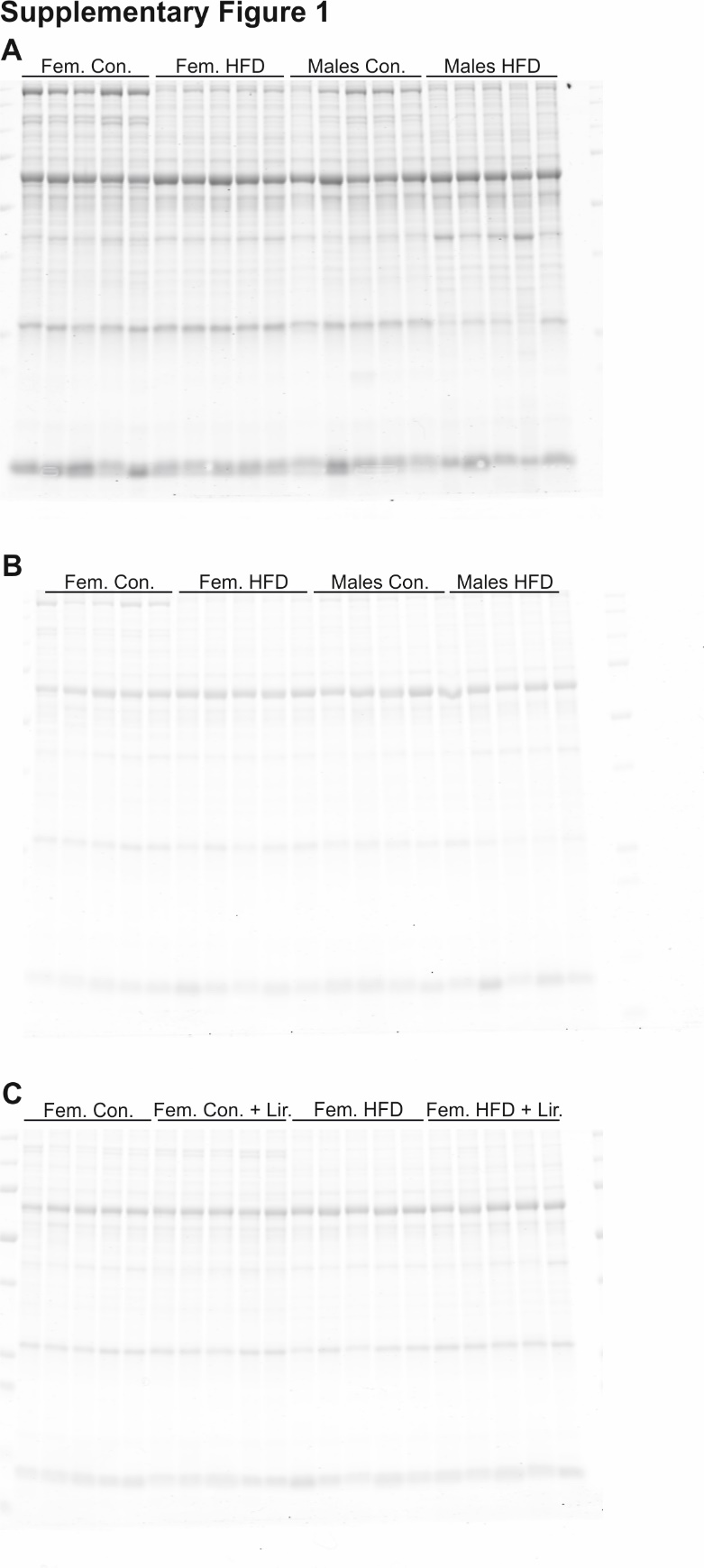


**SUPPLEMENTARY FIGURE 1:** Images of the Gelcode Coomassie Blue stained protein gels that was used to normalize the protein expression in adipose tissue. (A): Protein gel from female and male mice after 12 weeks of either control or HFD. (B): Protein gel from female and male mice after 24 weeks of either control or HFD. (C): Protein gel from female mice fed either control or HFD and with or without Liraglutide treatment.


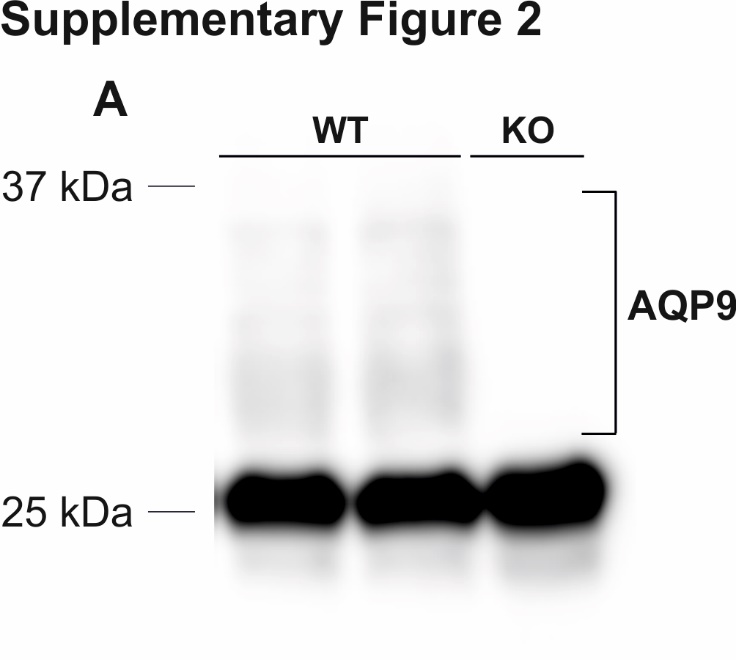


**SUPPLEMENTARY FIGURE 2:** Validation of which bands that are specific for AQP9 when using the AQP9 antibody (RA2674-685) for immunoblotting. (A): Immunoblot of AQP9 expression in mouse liver samples from two AQP9 wildtype mice (WT) and one AQP9 knockout (KO) mouse.
